# Supplementary material for: Threatening Facial Expressions Impact Goal-Directed Actions Only if Task-Relevant
Source: Brain Sci. 2020 Oct 29;10(11):794. doi: 10.3390/brainsci10110794 (PMC7694135; doi:10.3390/brainsci10110794)
Supplement: Supplementary file 1 [file brainsci-10-00794-s001.pdf]

**Table S1.** Results of the statistical analysis on reaction times (RTs). Post hoc tests (pairwise comparisons) had an adjusted alpha level corrected according to Bonferroni. Statistically significant results are reported in bold and italics. Bayes factors report the ratio of likelihood of the alternative hypothesis to the likelihood of the null hypothesis ($BF_{10}$). ANOVA, analysis of variance. Measures of size effects: $\eta_p^2$ for ANOVAs and Cohen's d for post hoc tests. Differences in the estimated marginal means ($M_{diff}$) are reported along with their 95% confidence interval (CI).

| **Four-way ANOVA of RT: Emotion (Anger, Happiness, Fear); Sex (F, M); AL (High, Medium, Low); Task (Emotion Discrimination Task, Gender Discrimination Task)** | | | | | | |
|---|---|---|---|---|---|---|
| | **Value of parameters** | ***p* values** | ***M**<sub></sub>* $M_{diff}$ | ***95% CI*** | **Effect Size** | **$BF_{10}$** |
| Main effect: **Emotion** | *F*[1.54, 76.82] = *51.81* | ***p<0.001*** | | | $\eta_p^2 = 0.51$ | $3.1*10^7$ |
| Post hoc Tests: | | | | | | |
| **Anger vs. Happiness** | *t*(111) = *8.46* | ***p<0.001*** | 19.27 | [13.63, 24.92] | *d = 0.80* | $4.4*10^8$ |
| **Anger vs. Fear** | *t*(111) = *6.80* | ***p<0.001*** | 15.07 | [9.58, 20.55] | *d = 0.64* | $1.2*10^6$ |
| **Happiness vs. Fear** | *t*(111) = *-3.14* | ***p=0.008*** | -4.21 | [-7.53, -0.89] | *d = 0.30* | 12.20 |
| Main effect: **Task** | *F*[1, 50] = *5.15* | ***p=0.028*** | 8.86 | [1.02, 16.70] | $\eta_p^2 = 0.093$ | 305.1 |
| Main effect: **AL** | *F*[2, 50] = *0.02* | *p=0.976* | | | $\eta_p^2 < 0.01$ | 0.21 |
| Main effect: **Sex** | *F*[1, 50] = *1.33* | *p=0.254* | | | $\eta_p^2 = 0.03$ | 0.60 |
| Interaction: **Emotion*Task** | *F*[1.77, 88.51] = *47.11* | ***p<0.001*** | | | $\eta_p^2 = 0.49$ | $5.7*10^7$ |
| Post hoc Tests: | | | | | | |
| **Emotion Task - Anger vs. Happiness** | *t*(55) = *10.08* | ***p<0.001*** | 37.02 | [27.92, 46.12] | *d = 1.35* | $1.9*10^{12}$ |
| **Emotion Task - Anger vs. Fear** | *t*(55) = *7.35* | ***p<0.001*** | 27.26 | [18.07, 36.45] | *d* = 0.98 | $9.3*10^7$ |
| **Emotion Task - Happiness vs. Fear** | *t*(55) = *-5.31* | ***p<0.001*** | -9.76 | [-14.31, -5.21] | *d = 0.71* | $1.8*10^4$ |
| **Gender Task - Anger vs. Happiness** | *t*(55) = *0.76* | *p=1* | 1.53 | [-3.48, 6.53] | *d = 0.10* | 0.20 |
| **Gender Task - Anger vs. Fear** | *t*(55) = *1.26* | *p=0.640* | 2.87 | [-2.75, 8.48] | *d = 0.17* | 0.21 |
| **Gender Task - Happiness vs. Fear** | *t*(55) = *0.60* | *p=1* | 1.34 | [-4.16, 6.85] | *d = 0.08* | 0.15 |
| **Emotion Task - Happiness vs. Gender Task - Happiness** | *t*(55) = *-1.58* | *p=0.121* | -6.67 | [-15.17, 1.83] | *d = 0.21* | 0.28 |
| **Emotion Task - Fear vs. Gender Task - Fear** | *t*(55) = *1.04* | *p=0.303* | 4.43 | [-4.12, 12.97] | *d = 0.14* | 0.29 |
| **Emotion Task - Anger vs. Gender Task - Anger** | *t*(55) = *5.92* | ***p<0.001*** | 28.83 | [19.05, 38.61] | *d = 0.79* | $2.6*10^5$ |
| Interaction: **Emotion*AL** | *F*[3.07, 76.82] = *0.54* | *p=0.657* | | | $\eta_p^2 = 0.02$ | 0.03 |
| Interaction: **Emotion*Sex** | *F*[1.54, 76.82] = *0.26* | *p=0.714* | | | $\eta_p^2 = 0.01$ | 0.06 |
| Interaction: **Task*AL** | *F*[2, 50] = *0.92* | *p=0.405* | | | $\eta_p^2 = 0.03$ | 0.42 |
| Interaction: **Task*Sex** | *F*[1, 50] = *1.57* | *p=0.217* | | | $\eta_p^2 = 0.03$ | 2.49 |
| Interaction: **AL*Sex** | *F*[2, 50] = *0.20* | *p=0.817* | | | $\eta_p^2 = 0.01$ | 0.33 |
| Interaction: **Emotion*Task*AL** | *F*[3.54, 88.51] = *0.26* | *p=0.883* | | | $\eta_p^2 = 0.01$ | 0.04 |
| Interaction: **Emotion*Task*Sex** | *F*[1.77, 88.51] = *2.80* | *p=0.073* | | | $\eta_p^2 = 0.05$ | 0.33 |

| | | | | | | |
|---|---|---|---|---|---|---|
| Interaction: **Emotion*AL*Sex** | $F[3.07, 76.82] = 0.51$ | $p=0.683$ | | | $\eta_p^2 = 0.02$ | 0.04 |
| Interaction: **Task*AL*Sex** | $F[2, 50] = 2.22$ | $p=0.119$ | | | $\eta_p^2 = 0.08$ | 39.2 |
| Interaction: **Emotion*Task*AL*Sex** | $F[3.54, 88.51] = 1.61$ | $p=0.184$ | | | $\eta_p^2 = 0.06$ | 0.92 |

**Table S2.** Results of the statistical analysis of movement times (MTs). Post hoc tests (pairwise comparisons) had an adjusted alpha level corrected according to Bonferroni. Statistically significant results are reported in bold and italics. Bayes factors report the ratio of likelihood of the alternative hypothesis to the likelihood of the null hypothesis ($BF_{10}$). ANOVA, analysis of variance. Measures of size effects: $\eta_p^2$ for ANOVAs and Cohen's d for post hoc tests. Differences of the estimated marginal means ($M_{diff}$) are reported along their 95% confidence interval (CI).

| **Four-way ANOVA of MT: Emotion (Anger, Happiness, Fear); Sex (F, M); AL (High, Medium, Low); Task (Emotion Discrimination Task, Gender Discrimination Task)** | | | | | | |
|---|---|---|---|---|---|---|
| | **Value of parameters** | ***p* values** | $M_{diff}$ | ***95%* CI** | **Effect Size** | $BF_{10}$ |
| Main effect: **Emotion** | $F[1.55, 77.29] = 16.64$ | ***p<0.001*** | | | $\eta_p^2 = 0.25$ | 2.82 |
| Post hoc Tests: | | | | | | |
| **Anger vs. Happiness** | $t(111) = 4.18$ | ***p<0.001*** | 8.10 | [3.31, 12.90] | $d = 0.40$ | 431 |
| **Anger vs. Fear** | $t(111) = 4.75$ | ***p<0.001*** | 8.31 | [3.98, 12.65] | $d = 0.45$ | 3120 |
| **Happiness vs. Fear** | $t(111) = 0.19$ | $p=1$ | 0.21 | [-2.61, 3.03] | $d = 0.02$ | 0.12 |
| Main effect: **Task** | $F[1, 50] = 3.34$ | $p=0.074$ | | | $\eta_p^2 = 0.06$ | 3.87 |
| Main effect: **AL** | $F[2, 50] = 0.18$ | $p=0.837$ | | | $\eta_p^2 = 0.01$ | 0.54 |
| Main effect: **Sex** | $F[1, 50] = 0.05$ | $p=0.822$ | | | $\eta_p^2 < 0.01$ | 0.61 |
| Interaction: **Emotion*Task** | $F[1.99, 99.48] = 16.54$ | ***p<0.001*** | | | $\eta_p^2 = 0.25$ | 1.49 |
| Post hoc Tests: | | | | | | |
| **Emotion Task - Anger vs. Happiness** | $t(55) = 4.84$ | ***p<0.001*** | 14.59 | [7.12, 22.05] | $d = 0.65$ | 3208 |
| **Emotion Task - Anger vs. Fear** | $t(55) = 5.63$ | ***p<0.001*** | 15.82 | [8.85, 22.78] | $d = 0.75$ | $2.0*10^4$ |
| **Emotion Task - Happiness vs. Fear** | $t(55) = 0.71$ | $p=1$ | 1.23 | [-3.06, 5.52] | $d = 0.10$ | 0.19 |
| **Gender Task - Anger vs. Happiness** | $t(55) = 0.98$ | $p=0.994$ | 1.62 | [-2.47, 5.71] | $d = 0.13$ | 0.16 |
| **Gender Task - Anger vs. Fear** | $t(55) = 0.55$ | $p=1$ | 0.81 | [-2.80, 4.42] | $d = 0.07$ | 0.17 |
| **Gender Task - Happiness vs. Fear** | $t(55) = -0.44$ | $p=1$ | -0.81 | [-5.38, 3.76] | $d = 0.06$ | 0.15 |
| **Emotion Task - Happiness vs. Gender Task - Happiness** | $t(55) = 1.06$ | $p=0.296$ | 5.54 | [-4.99, 16.08] | $d = 0.14$ | 0.16 |
| **Emotion Task - Fear vs. Gender Task - Fear** | $t(55) = 0.74$ | $p=0.462$ | 3.50 | [-5.98, 12.98] | $d = 0.10$ | 0.15 |
| **Emotion Task - Anger vs. Gender Task - Anger** | $t(55) = 3.17$ | ***p=0.003*** | 18.51 | [6.79, 30.23] | $d = 0.42$ | 5.67 |
| Interaction: **Emotion*AL** | $F[3.09, 77.29] = 0.16$ | $p=0.924$ | | | $\eta_p^2 < 0.01$ | 0.02 |
| Interaction: **Emotion*Sex** | $F[1.55, 77.29] = 0.09$ | $p=0.864$ | | | $\eta_p^2 < 0.01$ | 0.05 |
| Interaction: **Task*AL** | $F[2, 50] = 0.37$ | $p=0.692$ | | | $\eta_p^2 = 0.01$ | 0.08 |
| Interaction: **Task*Sex** | $F[1, 50] = 1.62$ | $p=0.209$ | | | $\eta_p^2 = 0.03$ | 1.40 |
| Interaction: **AL*Sex** | $F[2, 50] = 1.18$ | $p=0.315$ | | | $\eta_p^2 = 0.05$ | 0.84 |

| | | | | | | |
|---|---|---|---|---|---|---|
| Interaction: **Emotion*Task*AL** | *F*[3.98, 99.48] = *0.79* | *p=0.536* | | | $\eta_p^2$ = *0.03* | 0.02 |
| Interaction: **Emotion*Task*Sex** | *F*[1.99, 99.48] = *0.56* | *p=0.572* | | | $\eta_p^2$ = *0.01* | 0.09 |
| Interaction: **Emotion*AL*Sex** | *F*[3.09, 77.29] = *2.52* | *p=0.063* | | | $\eta_p^2$ = *0.09* | 0.06 |
| Interaction: **Task*AL*Sex** | *F*[2, 50] = *2.54* | *p=0.089* | | | $\eta_p^2$ = *0.09* | 429 |
| Interaction: **Emotion*Task*AL*Sex** | *F*[3.98, 99.48] = *1.98* | *p=0.104* | | | $\eta_p^2$ = *0.07* | 0.04 |

**Table S3.** Results of the statistical analysis on the percentage of mistakes. Post hoc tests (pairwise comparisons) had an adjusted alpha level corrected according to Bonferroni. Statistically significant results are reported in bold and italics. Bayes factors report the ratio of likelihood of the alternative hypothesis to the likelihood of the null hypothesis ($BF_{10}$). ANOVA, analysis of variance. Measures of size effects: $\eta_p^2$ for ANOVAs and Cohen's d for post hoc tests. Differences of the estimated marginal means ($M_{diff}$) are reported along their 95% confidence interval (CI). AL, arousal level.

| **Four-way ANOVA of Mistakes: Emotion (Anger, Happiness, Fear); Sex (F, M); AL (High, Medium, Low); Task (Emotion Discrimination Task, Gender Discrimination Task)** | | | | | | |
|---|---|---|---|---|---|---|
| | **Value of parameters** | ***p* values** | $M_{diff}$ | ***95%* CI** | **Effect Size** | $\mathbf{BF_{10}}$ |
| Main effect: **Emotion** | *F*[1.69, 84.78] = *37.44* | ***p<0.001*** | | | $\eta_p^2$ = *0.43* | $9.9*10^5$ |
| Post hoc Tests: | | | | | | |
| **Anger vs. Happiness** | *t*(111) = *7.01* | ***p<0.001*** | 3.54 | [2.29, 4.79] | *d = 0.66* | $4.9*10^4$ |
| **Anger vs. Fear** | *t*(111) = *6.56* | ***p<0.001*** | 3.27 | [2.03, 4.50] | *d = 0.62* | $1.0*10^5$ |
| **Happiness vs. Fear** | *t*(111) = *-0.79* | *p=1* | -0.27 | [-1.13, 0.58] | *d = 0.07* | 0.11 |
| Main effect: **Task** | *F*[1, 50] = *44.54* | ***p<0.001*** | 3.81 | [2.66, 4.96] | $\eta_p^2$ = *0.47* | $5.8*10^{13}$ |
| Main effect: **AL** | *F*[2, 50] = *3.97* | ***p=0.025*** | | | $\eta_p^2$ = *0.14* | 0.19 |
| Post hoc test: | | | | | | |
| **Low vs. Medium** | *t*(34) = *-1.24* | *p=0.663* | -1.03 | [-3.10, 1.03] | *d = -0.20* | 0.34 |
| **High vs. Medium** | *t*(34) = *1.65* | *p=0.314* | 1.42 | [-0.71, 3.54] | *d = 0.27* | 0.22 |
| **High vs. Low** | *t*(34) = *2.81* | ***p=0.021*** | 2.45 | [0.29, 4.61] | *d = 0.47* | 1.42 |
| Main effect: **Sex** | *F*[1, 50] = *5.27* | ***p=0.026*** | 1.60 | [0.20, 3.01] | $\eta_p^2$ = *0.10* | 0.44 |
| Interaction: **Emotion*Task** | *F*[1.56, 78.21] = *47.64* | ***p<0.001*** | | | $\eta_p^2$ = *0.49* | $8.3*10^{13}$ |
| Post hoc Tests: | | | | | | |
| **Emotion Task - Anger vs. Happiness** | *t*(55) = *7.58* | ***p<0.001*** | 7.55 | [5.09, 10.02] | *d = 0.72* | $9.7*10^7$ |
| **Emotion Task - Anger vs. Fear** | *t*(55) = *7.71* | ***p<0.001*** | 7.03 | [4.77, 9.29] | *d = 0.73* | $2.6*10^8$ |
| **Emotion Task - Happiness vs. Fear** | *t*(55) = *-0.97* | *p=1* | -0.53 | [-1.88, 0.82] | *d = 0.13* | 0.19 |
| **Gender Task - Anger vs. Happiness** | *t*(55) = *1.43* | *p=0.477* | -0.47 | [1.30, 0.35] | *d = 0.19* | 0.65 |
| **Gender Task - Anger vs. Fear** | *t*(55) = *1.38* | *p=0.521* | -0.49 | [1.38, 0.39] | *d = 0.18* | 0.32 |
| **Gender Task - Happiness vs. Fear** | *t*(55) = *0.05* | *p=1* | -0.02 | [-1.00, 0.96] | *d = 0.01* | 0.16 |
| **Emotion Task - Anger vs. Gender Task - Anger** | *t*(55) = *9.80* | ***p<0.001*** | 9.00 | [7.15, 10.84] | *d = 1.31* | $5.8*10^{11}$ |
| **Emotion Task - Happiness vs. Gender Task - Happiness** | *t*(55) = *1.30* | *p=0.198* | 0.97 | [-0.52, 2.46] | *d = 0.17* | 0.50 |
| **Emotion Task - Fear vs. Gender Task - Fear** | *t*(55) = *2.23* | ***p=0.030*** | 1.48 | [0.15, 2.80] | *d = 0.30* | 3.41 |
| Interaction: **Emotion*AL** | *F*[3.39, 84.78] = *0.46* | *p=0.737* | | | $\eta_p^2$ = *0.02* | 0.02 |
| Interaction: **Emotion*Sex** | *F*[1.69, 84.78] = *0.55* | *p=0.553* | | | $\eta_p^2$ = *0.01* | 0.08 |
| Interaction: **Task*AL** | *F*[2, 50] = *0.66* | *p=0.521* | | | $\eta_p^2$ = *0.03* | 0.17 |

| | | | | | | |
|---|---|---|---|---|---|---|
| Interaction: **Task*Sex** | $F[1, 50] = 0.04$ | $p=0.842$ | | | $\eta_p^2 < 0.01$ | 0.22 |
| Interaction: **AL*Sex** | $F[2, 50] = 5.85$ | **$p=0.005$** | | | $\eta_p^2 = 0.19$ | 5.97 |
| Post hoc Tests: | | | | | | |
| **Male - Low vs. Medium** | $t(50) = -0.10$ | $p=1$ | -0.10 | [-2.79, 2.58] | $d = 0.01$ | 0.19 |
| **Male - High vs. Medium** | $t(50) = 3.41$ | **$p=0.004$** | 4.31 | [1.17, 7.45] | $d = 0.45$ | 47.1 |
| **Male - High vs. Low** | $t(50) = 3.38$ | **$p=0.004$** | 4.42 | [1.18, 7.65] | $d = 0.45$ | 80.3 |
| **Female - Low vs. Medium** | $t(50) = -1.55$ | $p=0.384$ | -1.96 | [-5.10, 1.18] | $d = 0.21$ | 1.48 |
| **Female - High vs. Medium** | $t(50) = -1.28$ | $p=0.622$ | -1.48 | [-4.34, 1.39] | $d = 0.17$ | 0.60 |
| **Female - High vs. Low** | $t(50) = 0.42$ | $p=1$ | 0.48 | [-2.38, 3.35] | $d = 0.06$ | 0.23 |
| **Female - Low vs. Male - Low** | $t(50) = -0.76$ | $p=0.451$ | -0.91 | [-3.33, 1.50] | $d = 0.10$ | 0.33 |
| **Female - Medium vs. Male - Medium** | $t(50) = 0.82$ | $p=0.418$ | 0.94 | [-1.38, 3.27] | $d = 0.11$ | 0.30 |
| **Female - High vs. Male - High** | $t(50) = 3.83$ | **$p<0.001$** | 4.84 | [2.30, 7.39] | $d = 0.51$ | 406 |
| Interaction: **Emotion*Task*AL** | $F[3.13, 78.21] = 0.76$ | $p=0.527$ | | | $\eta_p^2 = 0.03$ | 0.10 |
| Interaction: **Emotion*Task*Sex** | $F[1.56, 78.21] = 0.74$ | $p=0.451$ | | | $\eta_p^2 = 0.01$ | 0.18 |
| Interaction: **Emotion*AL*Sex** | $F[3.39, 84.78] = 0.92$ | $p=0.443$ | | | $\eta_p^2 = 0.04$ | 0.12 |
| Interaction: **Task*AL*Sex** | $F[2, 50] = 0.51$ | $p=0.603$ | | | $\eta_p^2 = 0.02$ | 0.22 |
| Interaction: **Emotion*Task*AL*Sex** | $F[3.13, 78.21] = 0.32$ | $p=0.820$ | | | $\eta_p^2 = 0.01$ | 0.12 |

**Supplementary Table 4.** Correlations between the recognition score, and the mean values of behavioral parameters, characterizing the behavioral performance in the Emotion Discrimination task (average values of RTs, MTs, and rates of mistakes in Go-trials). For each behavioral parameter and each emotional facial expression, the value of the Spearman's correlation coefficient together with the corresponding p-values (2-tails) are reported.

| **Correlations between behavioral measures and the recognition scores** | | |
|---|---|---|
| | **Value of parameters** | **p values** |
| **RT vs. Recognition score (angry facial expressions)** | ϱ = 0.21 | p = 0.12 |
| **MT vs. Recognition score (angry facial expressions)** | ϱ = -0.11 | p = 0.42 |
| **Rates of Mistakes vs. Recognition score (angry facial expressions)** | ϱ = -0.23 | p = 0.09 |
| **RT vs. Recognition score (fearful facial expressions)** | ϱ = -0.01 | p = 0.93 |
| **MT vs. Recognition score (fearful facial expressions)** | ϱ = -0.07 | p = 0.62 |
| **Rates of Mistakes vs. Recognition score (fearful facial expressions)** | ϱ = 0.049 | p = 0.72 |
| **RT vs. Recognition score (happy facial expressions)** | ϱ = -0.12 | p = 0.37 |
| **MT vs. Recognition score (happy facial expressions)** | ϱ = -0.06 | p = 0.68 |
| **Rates of Mistakes vs. Recognition score (happy facial expressions)** | ϱ = 0.05 | p = 0.70 |
